# Inactivation of Zika Virus with Hydroxypropyl-Beta-Cyclodextrin

**DOI:** 10.3390/vaccines13010079

**Published:** 2025-01-16

**Authors:** Cory R. Hewitt, Nicholas J. Wixon, Arthur Gallegos, You Zhou, Victor C. Huber, M. Scott Killian

**Affiliations:** 1 Division of Basic Biomedical Sciences, Sanford School of Medicine, University of South Dakota, Vermillion, SD 57069, USA; chewitt@uchc.edu (C.R.H.); victor.huber@usd.edu (V.C.H.); 2 Microscopy Core Research Facility, Center for Biotechnology, University of Nebraska-Lincoln, Lincoln, NE 68588, USA; 3 Department of Public Health, School of Health Sciences, University of South Dakota, Vermillion, SD 57069, USA

**Keywords:** Zika virus, beta-cyclodextrin, envelope proteins, vaccine, antibody, immunogen, inactivation

## Abstract

**Background/Objectives**: Zika virus (ZIKV) infection is associated with life-threatening diseases in humans. To date, there are no available FDA-approved therapies or vaccines for the specific treatment or prevention of ZIKV infection. Variation in the ZIKV envelope protein (Env), along with its complex quaternary structure, presents challenges to synthetic approaches for developing an effective vaccine and broadly neutralizing antibodies (bnAbs). We hypothesized that beta-cyclodextrin (BCD) could be used to uniquely inactivate infectious ZIKV without disruption of Env. **Methods**: ZIKV was propagated in Vero cells and admixed with BCD. The BCD-treated ZIKV was evaluated for infectivity using immunofluorescence and quantitative RT-PCR (qRT-PCR) assays, for immunoreactivity in Western blots, structural integrity by electron microscopy, and immunogenicity in mice. **Results**: Here, we show that 200 mM BCD-treated ZIKV is non-infectious in cell culture, remains immunoreactive with an Env-specific antibody, retains its virion shape and size, and elicits the production of immunogen-specific antibodies in immunized mice. **Conclusions**: These results indicate that BCD can be used to safely inactivate ZIKV, and they provide insights for vaccine and antibody development.

## 1. Introduction

Zika virus (ZIKV), a member of the *Flaviviridae* family, is the causative agent of an emerging infectious disease that presents a global public health threat [1,2]. Other flaviviruses include dengue, yellow fever, and West Nile viruses. While the majority of ZIKV infections result from mosquito bites, transmission can also occur upon exposure to blood and bodily secretions, as well as following laboratory accidents [3]. Acute ZIKV infection is generally asymptomatic, but severe sequelae include congenital malformations and Guillain–Barre syndrome [4]. Notably, serious birth defects, such as microcephaly, occur in approximately 5% of babies born to women who experienced ZIKV infection during pregnancy [5]. Although several vaccines and candidate drugs are currently being evaluated, no definitive anti-ZIKV agents are available [1,6,7,8,9,10].

Effective vaccines are expected to induce a humoral immune response and the production of neutralizing antibodies (nAbs) specific for conserved epitopes present on the exterior surfaces of diverse strains of ZIKV that are essential for attachment and infectivity (i.e., broadly neutralizing antibodies; bnAbs). Exposed antigens on the infectious ZIKV particle include the envelope glycoprotein (E; 505 aa) and the membrane protein (M; 75 aa) [11]. The E and M proteins form dimeric heterodimer complexes that are arranged in a herringbone pattern [12]. Post-translational modifications of the ZIKV E protein, such as glycosylation and ubiquitination, contribute to the ability of the virus envelope to attach to a variety of host cell surface receptors (e.g., AXL, TIM-1, and DC-SIGN) [13,14]. Consequently, these structurally complex features of the native ZIKV E protein can be extremely difficult to reproduce using traditional vaccine design approaches.

Strategies for ZIKV vaccine development have focused on DNA and mRNA viral vectors, virus-like particles (VLPs), and the use of killed and attenuated live viruses [15]. While these various strategies can yield antibodies that neutralize ZIKV in vitro, their ability to protect animals against viral challenge is highly variable [10]. An important determinant of protection against viral challenge appears to be the ability of the antibodies to bind to mature ZIKV virions [16], suggesting that the quaternary structure of ZIKV E can be a crucial component [12]. Among vaccine development strategies, only the inactivation approach derives from native ZIKV E with certainty, as virus attenuation can be associated with alterations in envelope proteins [17,18]. Takeda’s purified inactivated ZIKV (PIZV) vaccine candidate retains virion integrity, as evidenced in electron micrographs, and has demonstrated safety and immunogenicity in a phase I clinical trial [19,20]. However, PIZV is manufactured using formaldehyde for virus inactivation, a procedure known to modify and crosslink proteins [21,22] and thereby likely altering native ZIKV E.

To address the challenges presented by the complex structure of the ZIKV envelope and its post-translational modification, we hypothesized that beta-cyclodextrin (BCD), a cyclic oligosaccharide that can act as a lipid chelator [23], could be used to render the virus non-infectious without disrupting the structural integrity of its surface antigens. Here, we report the successful derivation of an inactivated ZIKV particle immunogen that is replication-deficient, yet retains the natural quaternary envelope structure.

## 2. Materials and Methods

### 2.1. Virus Propagation in Cell Culture

ZIKV (VR-1843) was purchased from ATCC (Manassas, VA, USA) and propagated in African green monkey kidney epithelial (Vero) cells (ATCC; CRL-1586). This virus strain (also known as PRVABC59) was isolated from the blood of an infected human in Puerto Rico in 2015 and has been fully sequenced (GenBank: KU501215.1) [24]. All experiments with infectious ZIKV were conducted under biosafety level 2 (BSL2) containment procedures [25].

### 2.2. Inactivation of ZIKV

**Figure 1:**
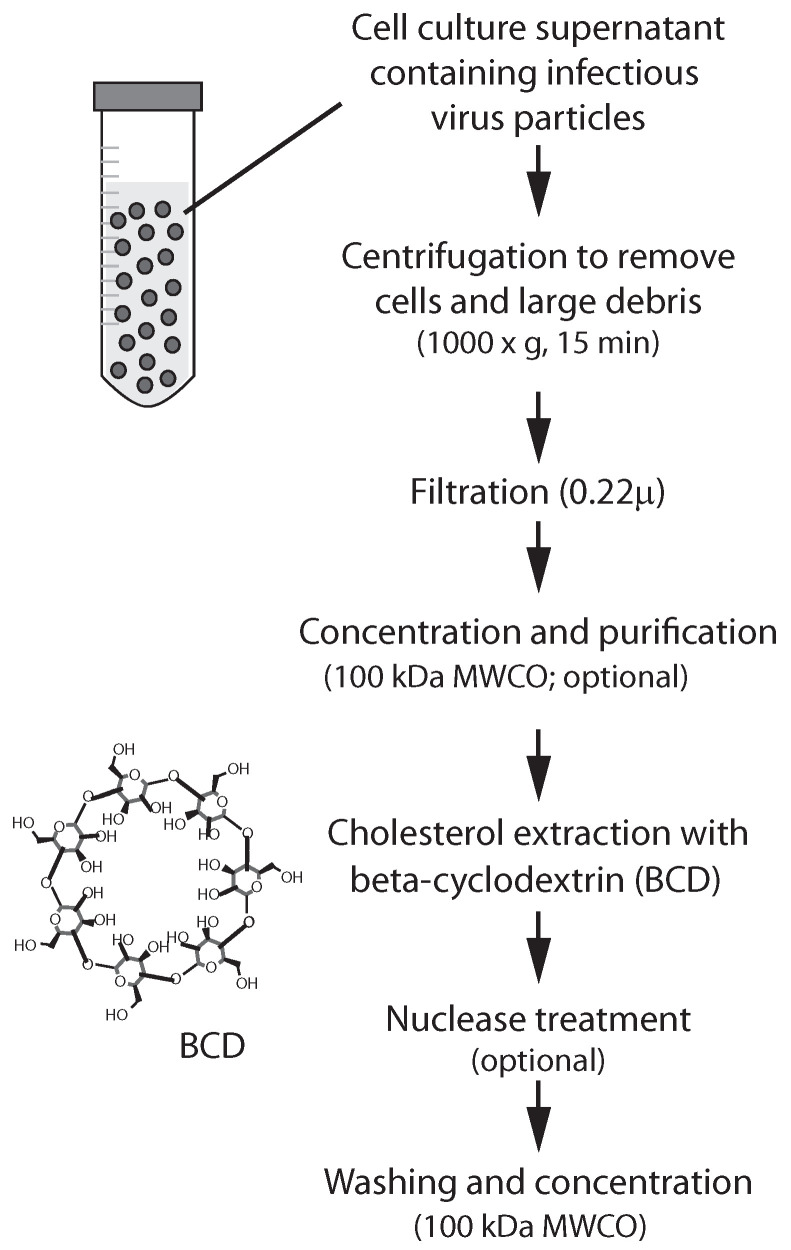
Treatment of ZIKV using beta cyclodextrin. Abbreviations: molecular weight cut-off (MWCO) filter.

To assess its ability to inactivate ZIKV, virus stocks were treated with varied concentrations of pharmaceutical grade 2-hydroxypropyl beta-cyclodextrin (BCD; Leternel) (Figure 1). Briefly, cell culture fluids containing infectious ZIKV were centrifuged (1000× *g* for 15 min) to remove cells and large debris. The resulting supernatant was filtered (0.22 µm) to remove smaller debris, and then concentrated using a 100 kDa molecular weight cutoff (MWCO) filter (Millipore, Billerica, MA, USA). ZIKV has a diameter of approximately 50 nanometers (nM) [12] and therefore will pass through a 0.22 m filter, but not a 100 kDa MWCO filter. Concentrated fluids were then admixed with varied amounts of a 200 millimolar (mM) BCD solution dissolved in Dulbecco’s phosphate-buffered saline without calcium or magnesium (PBS; Invitrogen, Carlsbad, CA, USA). BCD-treated fluids were washed with PBS using a 100 kDa MWCO filter. Parallel procedures were performed substituting PBS for BDC to generate fluids serving as a negative control for the BCD treatment.

### 2.3. Viral Plaque and Immunofluorescence Assays

Viral plaque assays were performed in accordance with routine procedures employing crystal violet staining for visual enhancement [26]. Immunofluorescence assays were performed to visually identify and enumerate ZIKV-infected cells. Briefly, for various experimental conditions, Vero cells were grown on glass coverslips, washed with PBS, and then fixed with 3.7% formaldehyde (Fisher, Waltham, MA, USA). The fixed cells were permeabilized and blocked for 1 h at room temperature with PBS containing 1% BSA and 0.3% Triton X-100.

The cells were then stained for 2 h at room temperature with a rabbit polyclonal anti-ZIKV E antibody (Genetex, Irvine, CA, USA; clone GTX133314). Afterwards, the cells were washed with PBS, blocked with 5% goat serum (Gibco, Grand Island, NY, USA) in PBS, and stained with a FITC-conjugated anti-rabbit-IgG antibody (Genetex) for 2 h at room temperature. Finally, the cells were washed and then mounted with Fluoroshield with DAPI (Genetex). Digital imaging was performed using a Zeiss Axio Imager M1 microscope with Axiovision (version 4.8) software.

### 2.4. Kinetic RT-PCR Assays

**Figure 2:**
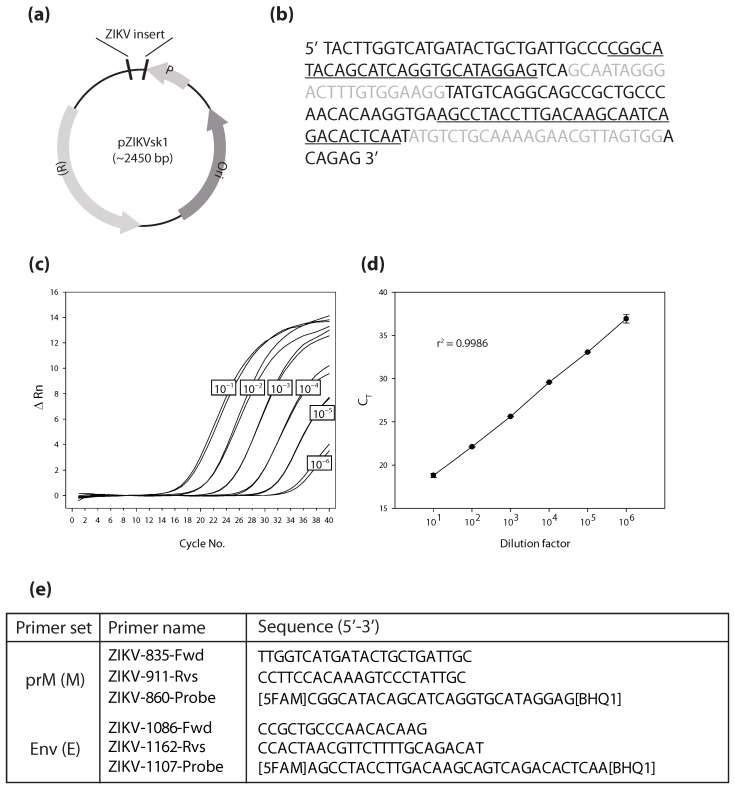
Assessment of ZIKV replication by kinetic RT-PCR. (**a**) A plasmid containing a ZIKV insert was constructed. (**b**) Denotes the specific insert that was utilized to encode parts of the ZIKV genome. Forward (Fwd) primers bind sequences in black, Reverse (Rvs) primers bind sequences shown in gray, and probes bind underlined sequences. (**c**) Log fold dilutions exhibit uniform kinetic PCR profiles with the Env primer set. (**d**) Graph demonstrating the correlation of PCR threshold cycle with plasmid dilution factor (Env primer set). (**e**) Two distinct primer sets were utilized to assess measurements of ZIKV E and M transcripts. Abbreviations: change in fluorescent signal (ΔRn), threshold cycle (C_T_).

To measure cell-free ZIKV levels in cell culture fluids, we used a quantitative RT-PCR assay. Following centrifugation (1000× *g* 10 min) to remove cells and large debris, RNA was extracted from cell culture supernatants (140 μL per sample) using a QIAamp Viral RNA Mini Kit (Qiagen, Germantown, MD, USA). A plasmid containing ZIKV-specific nucleotide sequences (pZIKVsk1) was designed in our laboratory (see Figure 2a) and commercially synthesized (Eurofins Genomics, Louisville, KY, USA). One-step RT-PCR reactions were performed using a QuantiNova Probe RT PCR kit (Qiagen) according to the manufacturer’s instructions and using a 7300 Real-Time PCR System (Applied Biosystems, Waltham, MA, USA). Previously described ZIKV-specific primers and probes were used to amplify and measure ZIKV M and E targets (see Figure 2e) [27].

### 2.5. Western Blotting

Virus and cell lysates were prepared by adding RIPA buffer (Genetex) to cell culture supernatants or cell pellets, respectively. The lysates were separated on 10% Bis-Tris mini gels (Invitrogen) and transferred to PVDF membranes using an iBlot apparatus (Invitrogen). Membranes were blocked overnight at 4 °C with BSA Blocker (Thermo Scientific, Waltham, MA, USA) and then incubated for 2 h at room temperature with a 1:1000 dilution rabbit polyclonal anti-ZIKV E antibody (Genetex; clone GTX133314). Afterwards, the membranes were washed with PBS containing 0.05% Tween-20, stained with an anti-rabbit-IgG antibody conjugated to HRP (Invitrogen) for 1 h at room temperature, washed again, incubated with a ECL Plus Western Blotting Substrate (Pierce, Waltham, MA, USA), and finally exposed to autorad film.

### 2.6. Electron Microscopy

To visualize ZIKV particles, transmission electron microscopy (TEM) was performed. The purified ZIKV particles, harvested with or without BCD treatment, were fixed in 2.5% glutaraldehyde in phosphate buffer. After two rinses in PBS, the samples were re-suspended in PBS. The virus suspensions were loaded onto formvar/carbon-coated grids and then processed for negative staining with 1% phosphotungstic acid. The stained samples were examined, and images were collected using a Hitachi H7500 transmission electron microscope.

### 2.7. Immunization of Mice

To evaluate the immunogenicity of the BCD-treated ZIKV, inoculation experiments were performed in mice. Adult (6–8-week-old) female BALB/cJ mice were purchased from Harlan Laboratories (Indianapolis, IN, USA). Hock immunizations [28] were performed at days 2 and 23 with 200 mM BCD-treated ZIKV in a 100 ul volume of PBS containing 2 mg/mL alum (General Chemical, Berkeley Heights, NJ, USA) as an adjuvant. Peripheral blood was collected by submandibular bleeds. All animal experiments were performed following the guidelines established and approved by the Animal Care and Use Committee at the University of South Dakota (Vermillion, SD, USA; IACUC 01-01-15-18D; approved 30 June 2017).

### 2.8. Dot Blots for Testing Antisera

To assess the immunoreactivity of the antisera from immunized mice with ZIKV, dot blot assays were performed. Briefly, BCD-inactivated ZIKV was spotted onto nitrocellulose strips. The strips were blocked with 5% BSA in PBS containing 0.05% Tween 20 (Fisher) for 2 h. Blocked strips were incubated for 2 h with mouse sera diluted in 5% BSA–PBS with gentle agitation. The strips were washed with PBS containing 0.05% Tween 20 and incubated with goat anti-mouse IgG H&L-Alexa Fluor 488 (Abcam, Waltham, MA, USA) at 1:200 dilution for 2 h at room temperature. Then, the strips were washed with PBS–0.05% Tween 20 and analyzed using an Odyssey CLx infrared imager (LI-COR, Lincoln, NE, USA).

### 2.9. Data Analysis

Data were compiled in Excel (Microsoft, Seattle, WA, USA) spreadsheets. When appropriate, experimental conditions were compared using the analysis of variance (ANOVA) or Student’s *t*-tests, with the Shapiro–Wilk test for normality. Statistical computations and graphs were prepared using SigmaPlot v11 (Grafiti, Palo Alto, CA, USA). *p* values <0.05 were considered to be statistically significant.

## 3. Results

### 3.1. Plaque and Immunofluorescence Assays for Assessment of ZIKV Replication In Vitro

Initial assessments of ZIKV replication were performed using both light microscopy and viral plaque assays (Supplementary data; Figure S1). While cytopathic effects [19] such as increased numbers of loosely attached cells were frequently observable (Figure S1a), our ZIKV isolate did not always produce visible clearings in Vero cells cultured to confluency and therefore conventional plaque assays did not reliably distinguish between infected and uninfected cell cultures (Figure S1b). However, ZIKV-infected cells were distinguishable in immunofluorescence assays as evidenced by positive staining for the ZIKV Env protein (Figure S1c). In addition, supernatants from infected Vero cell cultures exhibited ZIKV RNA levels that markedly increased (>500-fold) during the 3-day period following the acute infection procedure, further demonstrating robust ZIKV replication.

### 3.2. Quantitative RT-PCR Assay for Measurement of ZIKV

To develop a quantitative assay for the measurement of ZIKV levels in cell culture fluids (Figure 2), we successfully constructed a plasmid (pZIKVsk1; Figure 2a) containing an insertion of nucleotide sequences specific to the ZIKV genome and amplifiable with previously described PCR primer/probe sets specific for ZIKV M (prM) and E (Env) sequences (Figure 2a,b,e) [17]. Serial log-fold dilutions of pZIKVsk1 exhibited uniformly spaced kinetic PCR amplification profiles using primer set E (Figure 2c), and the PCR threshold cycle was tightly correlated (r2 = 0.9986) with the plasmid dilution factor (Figure 2d). Similar results were obtained with PCR amplification using the ZIKV M primer set. These results establish the utility of an improved quantitative RT-PCR (qRT-PCR) assay for measuring ZIKV levels in cell culture fluids.

### 3.3. Effects of BCD Treatment on ZIKV

**Figure 3:**
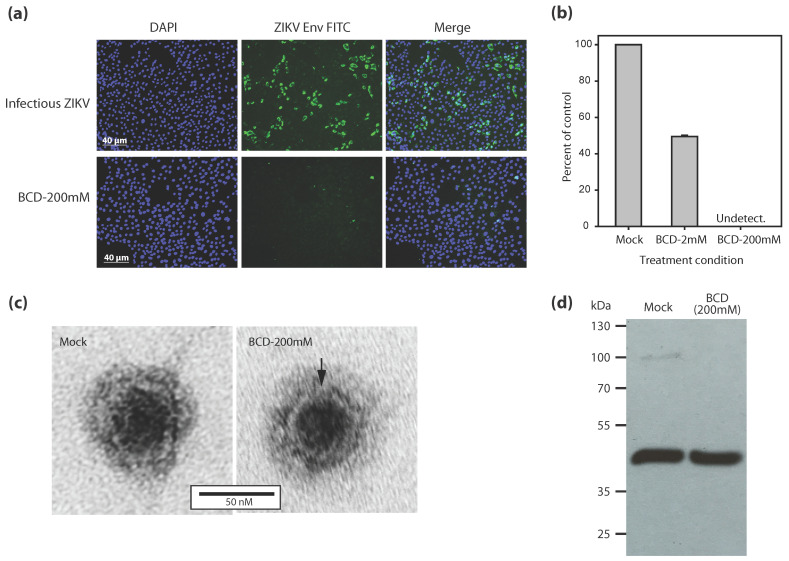
Inactivation of ZIKV using beta cyclodextrin. (**a**) Vero cells were exposed to mock-treated (**upper**) and BCD-treated (**lower**) ZIKV and the cells were then evaluated for virus production by fluorescent microscopy. (**b**) ZIKV levels in Vero cell cultures exposed to ZIKV treated with various concentrations of BCD. (**c**) Electron microscopy images of mock- and BCD-treated ZIKV particles. (**d**) Western blot images of mock- and BCD-treated ZIKV. Abbreviations: 4′,6-diamidino-2-phenylindole (DAPI), fluorescein isothiocyanate (FITC). The original Western blot figure can be found in Supplementary File S1.

The effects of BCD treatment on ZIKV were evaluated in immunofluorescence assays, electron micrographs, and Western blots (Figure 3). Immunofluorescence assays revealed a markedly visible reduction in the frequency of cells staining positive for ZIKV Env in the cultures infected with 200 mM BCD-treated ZIKV (Figure 3a). Enumeration of the percentage of fluorescent positive cells relative to PBS-treated controls revealed a dose-dependent effect of BCD treatment on ZIKV Env levels (Figure 3b). Treatment with 2 mM BCD was observed to result in approximately a 50% reduction in the percentage of infected cells expressing ZIKV Env, and this protein was virtually undetectable with 200 mM BCD treatment. Although some background fluorescence was evident with the BCD-treated virus, the fluorescence did not appear to be concentrated in the cytoplasm or at the cell surface. Also, the fluorescence levels in the BCD-treated virus cultures were similar to those observed with uninfected cultures (see Figure S1c). Importantly, the reduction in ZIKV Env levels in the Vero cell cultures was not due to the depletion of Env from the virus inoculate, as mock- and BCD-treated virus stocks exhibited very similar Env levels in Western blot analyses (Figure 3d).

For visual comparisons at high magnification, transmission electron microscopy was performed (Figure 3c). The mock (PBS)- and BCD-treated virus preparations featured the presence of distinct spherical populations of intact virus particles that were approximately 50–70 nm in diameter, consistent with prior size estimates [15]. Most of the viruses in the mock- and 200 mM BCD-treated samples exhibited some permeability as evidenced by internal dark/dense regions where the dye penetrated the particles and presumably due to the use formalin fixative. These results are consistent with prior observations of BCD-treated HIV particles [20,21] and support the premise that BCD inactivates ZIKV by depleting cholesterol from its lipid membrane without complete disintegration of the virus membrane and without substantial reductions in Env [22].

**Figure 4:**
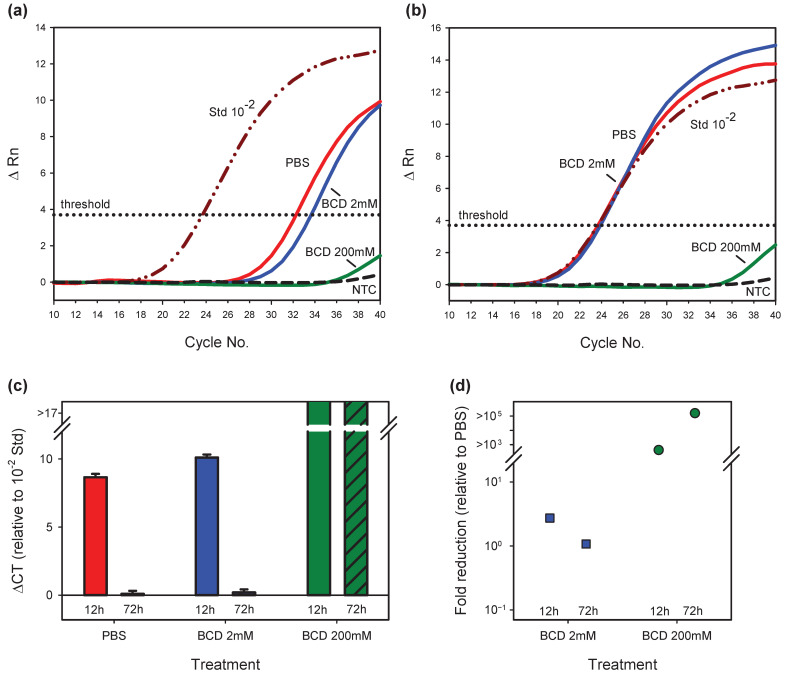
qRT-PCR analysis of cultures infected with BCD-treated ZIKV. qRT-PCR was performed on cell culture supernatants at (**a**) 12 h and (**b**) 72 h post infection with PBS, 2 mM, or 200 mM BCD-treated ZIKV. (**c**) For each treatment condition (PBS in red, 2 mM BCD in blue, 200 mM BCD in green; 12 h in solid fills, 72 h in hatched fills), differences in the CT values were computed relative to the CT of the 10^−2^ dilution of the pZIKVsk1 plasmid. (**d**) Fold reduction values were computed for the 12 h and 72 h BCD-treated conditions relative to the PBS-treatment condition. Values above the breaks in the plots (c and d) indicate values below the threshold of quantitation. Abbreviations: change in fluorescent signal (ΔRn), threshold cycle (C_T_).

In addition, the effects of BCD treatment on ZIKV replication in vitro were enumerated by qRT-PCR (Figure 4). At 12 h post infection (Figure 4a,c,d) and in comparison to treatment with PBS (negative control), 2 mM BCD reduced ZIKV levels by approximately 2-fold, while 200 mM BCD reduced ZIKV levels by >2 logs and below the threshold of quantitation. At 72 h post infection (Figure 4b–d) and in comparison to treatment with PBS, the effect of 2 mM BCD on ZIKV levels was negligible, while 200 mM BCD reduced ZIKV levels by >5 logs and below the threshold of quantitation. Importantly, relative to the 10^−2^ dilution of pZIKVsk1 (Figure 4c), cell cultures infected with the PBS- and 2 mM BCD-treated ZIKV exhibited 8–10 doublings of virus levels during the 12 h to 72 h interval, while cultures exposed to the 200 mM-treated ZIKV exhibited no appreciable increase in virus levels. These results strongly indicate that 200 mM BCD treatment renders ZIKV non-infectious in vitro.

### 3.4. Immunization of Mice with BCD-Treated ZIKV

**Figure 5:**
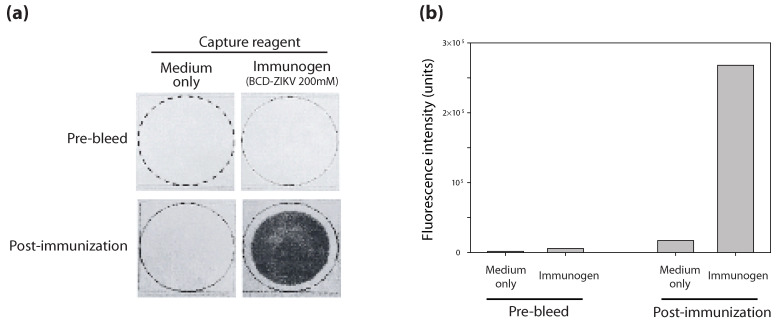
Antibody production in mice immunized with BCD-treated ZIKV. (**a**) Dot blots showing the immunoreactivity of serum collected from a mouse pre- and post-immunization with BCD-treated ZIKV. (**b**) Relative antibody concentrations were calculated based on the fluorescence intensities of the spots.

Holistic and immunological effects were assessed in mice (n = 3) inoculated with 200 mM BCD-treated ZIKV (Figure 5). None of the immunized mice exhibited any signs of pathogenic viral infection, including lethargy, significant weight loss, or death. Sera collected from the mice inoculated with 200 mM BCD-treated ZIKV, but not those collected prior to immunization (pre-bleed), exhibited strong cross-reactivity with the inoculum (Figure 5a,b). Also, sera collected from the mice did not cross react with cell culture medium. These results demonstrate that BCD-treated ZIKV is immunogenic in mice without pathogenic effects.

## 4. Discussion

Few studies have assessed methods for inactivating ZIKV, and to our knowledge, this is the first study to establish a procedure (Figure 1) using BCD to diminish the infectivity of ZIKV. BCD formulations are widely used in the pharmaceutical, cosmetics, and food industries and have been demonstrated safe for certain human applications [29,30]. Moreover, BCD is inexpensive, BCD solutions are simple to prepare in the laboratory, and pharmaceutical-grade BCD is commercially available, making the use of BCD scalable and feasible for various research and clinical applications.

In the present study, the infectivity of ZIKV was reduced by 50% with 2 mM BCD and by greater than 99% with 200 mM BCD in immunofluorescence assays (Figure 3). By qRT-PCR, we showed that 200 mM BCD reduces ZIKV levels by >5 logs in 72 h Vero cell cultures (Figure 4). Our findings that ZIKV can be substantially inactivated with BCD treatment in a dose-dependent manner are consistent with prior reports on HIV, SARS-CoV-2, and other viruses [31,32,33,34]. Importantly, BCD treatment results in a mechanism of virus inactivation that is distinct from those of common agents that crosslink or denature exposed proteins (i.e., modifying potential critical epitopes needed for the generation of broadly neutralizing antibodies), such as formaldehydes or heat [35,36,37]. Cyclodextrins are believed to function in virus inactivation through the depletion of membrane cholesterol and the disruption of lipid rafts without modification of surface proteins [23,31,32,34]. Our observations with BCD-treated ZIKV can suggest that ZIKV, like HIV, requires membrane-associated cholesterol for attachment and entry into host cells [38]. Moreover, by forming pores in the viral membrane, BCD can cause detrimental changes in the pH or osmolarity of the virus, or potentially enable seepage of nucleic acids and internal proteins.

Vaccination can be effective for the prevention of mosquito-borne and sexually transmitted ZIKV infection. Candidate vaccines are being actively developed and evaluated, and include recombinant yellow fever virus- and adenovirus-based vaccines containing ZIKV E [7]. However, recombinant viruses can present a variety of safety and efficacy concerns, making the production of a natural inactivated ZIKV immunogen an attractive approach. Our results demonstrate that 200 mM BCD-inactivated ZIKV particles retain immunoreactive Env (Figure 3) and elicit immunogen-specific antibodies in immunized mice (Figure 5). Also, our results demonstrate that BCD-inactivated ZIKV particles can be safely used as ‘bait’ in immunoassays to capture and enumerate ZIKV-specific antibodies. In this application, BCD-inactivated ZIKV could facilitate the identification of therapeutic bnAbs or the evaluation of vaccine candidates to induce antibodies specific for unmodified ZIKV Env. Because the mechanism of action of BCD involves the general depletion of cholesterol from lipid membranes and is not virus strain-specific, BCD treatment could be applied to divergent ZIKV strains to create vaccine ‘cocktails’.

The present studies leave several important questions for vaccine development unanswered. First, does BCD treatment indirectly modify ZIKV Env proteins, perhaps mediated by cholesterol depletion causing the disruption of hydrophobic interactions or van der Waals forces? Our electron migrographs (Figure 4c) demonstrate that the BCD-treated ZIKV general structure remains intact, but lack the resolution required for fine inspection of Env protein structure. Second, does BCD-treated ZIKV feature Env proteins that structurally differ from the Env proteins derived from other inactivation procedures (e.g., formaldehyde or heat)? Both of these questions are important because they can further establish the extent to which various virus inactivation procedures disrupt the native ZIKV Env structure and thereby modify the epitopes required to elicit broadly neutralizing antibodies. Answers to these first two questions will likely require the use of X-ray crystallography. Third, does inoculation with BCD-treated ZIKV elicit the production of unique broadly neutralizing antibodies? Our experiments in mice (Figure 5) show that inoculation with BCD-treated ZIKV can safely elicit the production of antibodies specific for the inoculum, which is a necessary but not sufficient requirement. Demonstration of cross-strain activity in virus neutralization assays is a reasonable next step toward answering this question. And ultimately, does a vaccine composed of BCD-treated ZIKV protect humans against pathogenic ZIKV infection?

In summary, our findings provide insight for several distinct aspects of ZIKV control. BCD can be used to inactivate or ‘sterilize’ ZIKV in cell culture fluids and presumably other liquids and blood products. BCD-inactivated ZIKV can be used as a safe substrate in assays for the evaluation of vaccine and antibody candidates that require intact Env proteins. Topical products containing BCD can possibly be used as prophylaxis to protect against sexually transmitted ZIKV infection. In addition, BCD-inactivated ZIKV is immunogenic in mice, raising the possibility that this product could be directly used to produce an effective vaccine or novel therapeutic antibodies. Additional studies are needed to further characterize the molecular structure of Env proteins in BCD-treated ZIKV, to determine whether BCD-treated ZIKV can elicit bnAbs, and ultimately the efficacy of a BCD-inactivated ZIKV vaccine.

## Data Availability

The original contributions presented in this study are included in the article. Further inquiries can be directed to the corresponding author.

## Supplementary Materials

The following supporting information can be downloaded at: https://www.mdpi.com/article/10.3390/vaccines13010079/s1, Figure S1: Assessment of ZIKV replication in microscopic and immunoassays; File S1: The original Western blot figure.
