# Supplementary material for: Inactivation of Zika Virus with Hydroxypropyl-Beta-Cyclodextrin
Source: Vaccines (Basel). 2025 Jan 16;13(1):79. doi: 10.3390/vaccines13010079 (PMC11769224; doi:10.3390/vaccines13010079)
Supplement: Supplementary file 1 [file vaccines-13-00079-s001.zip › vaccines-3289601-Figure S1.pdf]

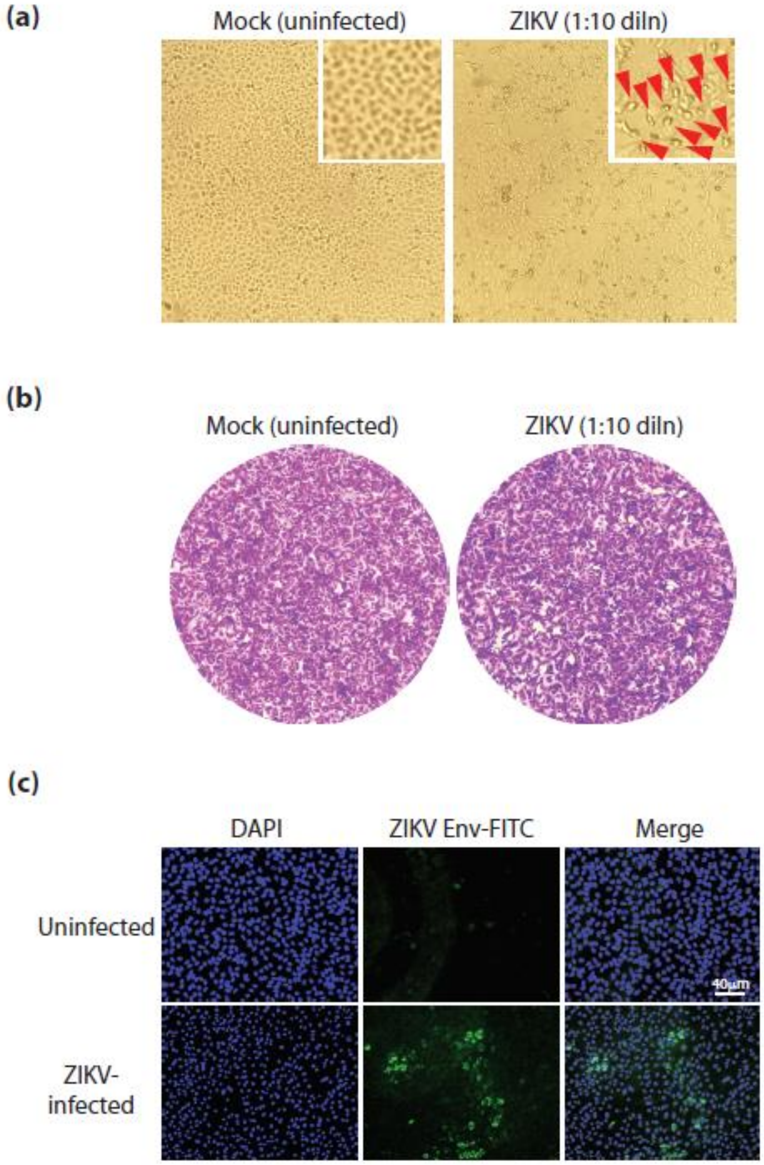

Figure S1. Assessment of ZIKV replication in microscopic and immunoassays. **(a)** Light microscopy images of plaque assays that were performed on mock- and ZIKV-infected Vero cells 3-4 days following infection. Arrows identify observable cytopathic effects. A 1:10 dilution (1:10 diln) of the ZIKV stock was used to infect the Vero cells. **(b)** Light microscopy images of stained cells in plaque assays demonstrating confluence of cells in both mock and ZIKV cell cultures. **(c)** Immunofluorescence (DAPI and ZIKV Env FITC) staining of uninfected- and ZIKV-infected Vero cells.
