# Supplementary material for: Inactivation of Zika Virus with Hydroxypropyl-Beta-Cyclodextrin
Source: Vaccines (Basel). 2025 Jan 16;13(1):79. doi: 10.3390/vaccines13010079 (PMC11769224; doi:10.3390/vaccines13010079)

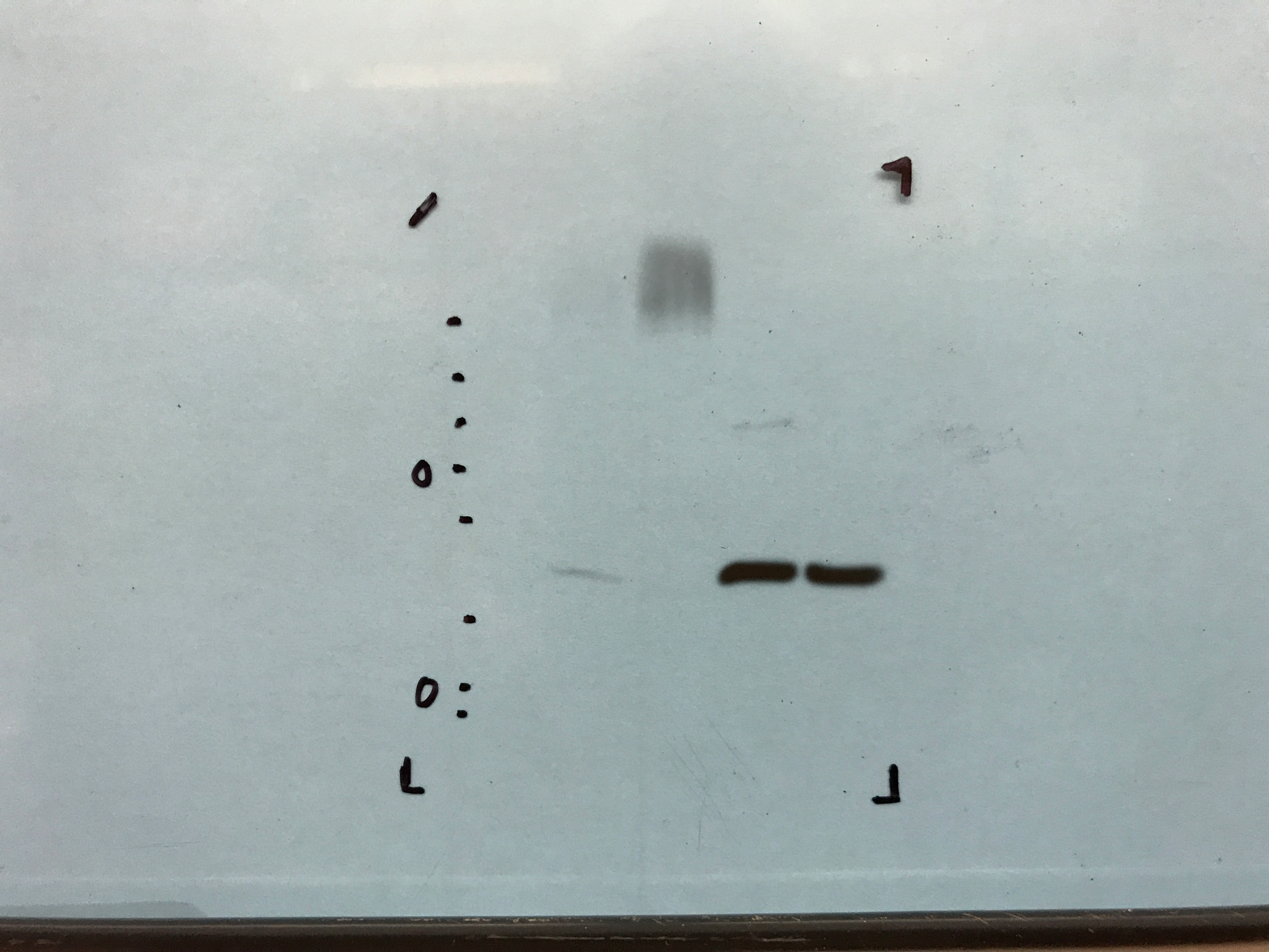

Supplement: Supplementary file 1 [file vaccines-13-00079-s001.zip › vaccines-3289601-File S1. The original Western blot figure.pdf]
